# Assessment of Factors Associated With Community-Acquired Extended-Spectrum β-Lactamase–Producing *Escherichia coli* Urinary Tract Infections in France

**DOI:** 10.1001/jamanetworkopen.2022.32679

**Published:** 2022-09-21

**Authors:** Adeline Paumier, Antoine Asquier-Khati, Sonia Thibaut, Thomas Coeffic, Olivier Lemenand, Stéphanie Larramendy, Brice Leclère, Jocelyne Caillon, David Boutoille, Gabriel Birgand

**Affiliations:** 1 Centre d’Appui à la Prévention des Infections Associées aux Soins des Pays de la Loire, Centre Hospitalier Universitaire (CHU)–Le Tourville, Nantes, France; 2 Department of General Practice, Faculty of Medicine, University of Nantes, Nantes, France; 3 French National Surveillance System of Antimicrobial Resistance in Primary Care and Nursing Homes, PRIMO, CHU–Le Tourville, Nantes, France; 4 Department of Infectious Diseases, University Hospital of Nantes and Centre d’Investigation Clinique 1413, Institut National de la Santé et de la Recherche Médicale, Nantes, France; 5 Department of Medical Evaluation and Epidemiology, CHU Nantes, Pays de la Loire, Nantes, France; 6 National Institute for Health Research Health Protection Research Unit in Healthcare Associated Infection and Antimicrobial Resistance at Imperial College London, Hammersmith Campus, London, United Kingdom

## Abstract

**Importance:**

Extended-spectrum β-lactamase (ESBL)–producing *Escherichia coli* is considered a leading pathogen contributing to the global burden of antimicrobial resistance.

**Objective:**

To better understand factors associated with the heterogeneity of community-acquired ESBL-producing *E coli* urinary tract infections (UTIs) in France.

**Design, Setting, and Participants:**

This cross-sectional study performed from January 1 to December 31, 2021, was based on data collected via PRIMO (Surveillance and Prevention of Antimicrobial Resistance in Primary Care and Nursing Homes), a nationwide clinical laboratory surveillance system in France. Strains of *E coli* isolated from community urine samples from January 1 to December 31, 2019, from 59 administrative departments of metropolitan France were included.

**Main Outcomes and Measures:**

Quasi-Poisson regression models were used to assess the associations between several ecological factors available on government and administration websites between 2010 and 2020 (demographic population structure, living conditions, baseline health care services, antibiotic consumptions, economic indicators, animal farming density, and environmental characteristics) and the number of ESBL-producing *E coli* strains isolated from urine samples of individuals with community-acquired UTI in 2019.

**Results:**

Among 444 281 *E coli* isolates from urine samples tested in 1013 laboratories, the mean prevalence of ESBL-producing *E coli* was 3.0% (range, 1.4%-8.8%). In an adjusted model, the number of community-acquired ESBL-producing *E coli* UTIs in each department was positively associated with the percentage of children younger than 5 years (adjusted β_1_ coefficient, 0.112 [95% CI, 0.040-0.185]; *P* = .004), overcrowded households (adjusted β_1_ coefficient, 0.049 [95% CI, 0.034 to 0.062]; *P* < .001), consumption of fluoroquinolones (adjusted β_1_ coefficient, 0.002 [95% CI, 0.001-0.002]; *P* < .001), and tetracyclines (adjusted β_1_ coefficient, 0.0002 [0.00004 to 0.00039]; *P* = .02), and poultry density (adjusted β_1_ coefficient, 0.0001 [95% CI, 0.0001-0.0002]; *P* < .001). The social deprivation index (adjusted β_1_ coefficient, −0.115 [95% CI, −0.165 to −0.064]; *P* < .001) and the proportion of water surface area (adjusted β_1_ coefficient, −0.052 [−0.081 to −0.024]; *P* = .001) were negatively associated with a higher number of community-acquired ESBL-producing *E coli* UTIs.

**Conclusions and Relevance:**

The findings of this cross-sectional study suggest that multiple human health, animal health, and environmental factors are associated with the occurence of community-acquired ESBL *E coli* UTI. Strategies to mitigate ESBL in the community should follow the One Health approach and address the role played by fluoroquinolones, tetracycline use, poultry density, overcrowded households, and preschool-aged children.

## Key Points

**Question:**

Which health care, socioeconomic, environmental, and agricultural factors are associated with community-acquired extended-spectrum β-lactamase (ESBL)–producing *Escherichia coli* urinary tract infections (UTIs) in France?

**Findings:**

In this cross-sectional study, ESBL-producing *E coli* isolated from urine samples of individuals with community-acquired UTI was associated with the local percentage of children younger than 5 years, overcrowded households, human consumption of fluoroquinolones and tetracyclines, and poultry density in administrative departments of metropolitan France.

**Meaning:**

These findings suggest that various population-level exposures are associated with increased likelihood of community-acquired ESBL-producing *E coli* UTI.

## Teaser

This cross-sectional study examines the ecological factors associated with community-acquired extended-spectrum β-lactamase–producing *Escherichia coli* urinary tract infections in France.

## Introduction

Initially confined to the health care environment, infections caused by extended-spectrum β-lactamase (ESBL)–producing *Escherichia coli* among patients from the community have become common in many countries.^1^ Risk factors for community-acquired ESBL-producing *E coli* urinary tract infection (UTI) are still poorly understood. Previous studies^2,3^ have suggested that both individual and ecological determinants are associated with the occurrence of community-acquired ESBL-producing *E coli* UTI. Antibiotic treatments in the preceding months and recent exposure to hospital and health care activities (eg, urinary catheter) are the most frequently cited individual factors.^4^ From an epidemiological perspective, multiple sources of acquisition and transmission pathways for community-acquired ESBL-producing *E coli* have been described. In the general population, ESBL-producing *E coli* infection is associated with social deprivation,^5^ recent international travel in endemic countries,^6^ or transmission among members of overcrowded households.^7^ In addition, the presence of ESBL-producing *E coli* in livestock—including poultry, pigs, and cattle—has been associated with an increased risk of colonization and subsequent infection among humans living in close proximity to livestock.^8,9^ Finally, the risk of community-acquired ESBL-producing *E coli* UTI has also been associated with ESBL detected in waterways and aquatic environments close to health care centers,^10^ wastewater,^11^ or agricultural lands^12^ that may be contaminated through the spreading of livestock manure containing both ESBL and antibiotics.

The assessment of factors associated with the spatial heterogeneity of community-acquired ESBL-producing *E coli* UTI in France may highlight new potential areas of interest for the management of antimicrobial resistance in the community. We performed a cross-sectional study using publicly available data on government and administration websites to study the ecological factors (population structure, living conditions, health care services, economic indicators, and agricultural and environmental characteristics) associated with community-acquired ESBL-producing *E coli* UTI across the country.

## Methods

### Study Design and Setting

This cross-sectional study used retrospective epidemiological and microbiological data collected via PRIMO (Surveillance and Prevention of Antimicrobial Resistance in Primary Care and Nursing Homes), a nationwide clinical laboratory surveillance system, for administrative departments in France. We restricted the data set to clinical laboratories that provided data during the entire 2019 calendar year (January 1 to December 31, 2019). Clinical laboratories involved in the PRIMO nationwide network participated on a voluntary basis. There is no current centralized database for pathology results at the national level in France. Because the analysis was performed using anonymized surveillance data, ethical consent was not required according to the French Data Protection Act. The database was accredited by the French National Data Protection Commission, and the fully anonymized data waiver for informed consent of study participants was applied. This study followed the Strengthening the Reporting of Observational Studies in Epidemiology (STROBE) reporting guideline.

### Study Population

The isolates were identified by a search in the PRIMO surveillance database for urine samples positive for *E coli* in primary care. In French guidelines, urine sampling is recommended for patients at risk of UTI (eg, pregnancy, anomalies of urinary tract, older than 75 years, immunosuppression, renal failure) or with complicated UTI.^13^ In 2019, the PRIMO surveillance system collected all routine antibiotic resistance data on *Enterobacterales* isolates from 1013 clinical laboratories distributed throughout France (eFigure 1 in Supplement 1). Each participating laboratory gathered individual data from clinical samples prescribed by clinicians in general practice for community patients and submitted the results to a central database located at the Nantes University Hospital, Nantes, France. Data were also collected regarding patient age and sex, the type of specimen, and the microorganism isolated from the specimen (species, full antibiograms, results from phenotypic analysis). To limit uncertainty in the estimate due to the small size, a minimum number of *E coli*–positive urine test results per administrative department was determined. To do so, the expected rate of ESBL-producing *E coli* in urine samples was estimated at 3.5% based on literature data.^14^ With an α risk set at 5%, we considered that a minimum number of 325 samples per department was needed to estimate the rate of urine samples positive for ESBL-producing *E coli* with a margin of 2.0%.

### ESBL-Producing *E coli* Isolates Included in Administrative Departments

For this study, only urine samples yielding *E coli* growth in the community setting were included. To avoid duplicates, only the first isolate with a same susceptibility pattern cultured in a single clinical laboratory and from an individual with the same date of birth and sex was considered for the current analysis. The microbiological data included the antibiotic susceptibility patterns. Antimicrobial susceptibility and the production of ESBL were consistently tested in the participating laboratories according to the European Committee on Antimicrobial Susceptibility Testing guidelines.^15^ The microbiological and epidemiological data available for each isolate allowed the stratification by administrative department, patient age and sex, and type of specimen.

### Ecological Factors and Data Collection

Ecological factors potentially associated with the number of community-acquired ESBL-producing *E coli* UTIs were selected based on risk factors and assumptions commonly described in the literature and publicly available data (Table 1). The selected sociodemographic factors were sex, age,^16^ and deprivation index.^17^ The composite deprivation index measures socioeconomic disadvantage based on 4 variables: the rate of unemployment in the active population aged 15 to 64 years, the rate of manual labor workers (skilled or unskilled, involving manufacturing, warehousing, mining, excavation, and many other types of physical work), the rate of high school graduates, and the median household income per consumption unit. The densities of hospitals^18^ and nursing homes^19^ (in beds per square kilometer) by department in 2019 were used as collective health care–related indicators. The number of antibiotic prescribers (clinicians in general practice and dentists) per 100 000 inhabitants was used as an individual health care–related indicator.^20^ We extracted the quantities of antibiotics delivered to the community for each department from 2015 to 2019.^21^ The total quantity of antibiotics delivered was calculated for each administrative department as the mean daily defined doses per 1000 inhabitants per year. The quantities delivered were also calculated by antibiotic classes for the same period.

**Table 1.** Definition of Local Characteristics of the French Administrative Departments Analyzed^a^

Characteristic	Data source	Year
**Health care–related**		
Antibiotics, No. of DDDs/1000 inhabitants per y		
All	Geodes	2015-2019
β-Lactams	Geodes	2015-2019
Broad-spectrum penicillin	Geodes	2015-2019
Penicillin associations	Geodes	2015-2019
Cephalosporins	Geodes	2015-2019
2GC	Geodes	2015-2019
3GC and/or 4GC	Geodes	2015-2019
Macrolides	Geodes	2015-2019
Fluoroquinolones	Geodes	2015-2019
Sulfonamides and trimethoprim	Geodes	2015-2019
Tetracyclines	Geodes	2015-2019
Associations and others	Geodes	2015-2019
Density of antibiotic prescribers, No./100 000 inhabitants	DREES	2019
Density of hospital beds, No./total area	SAE	2019
Density of nursing homes, No./total area	FINESS	2020
**Sociodemographic**		
Population density, No. of inhabitants/total area	INSEE	2019
Proportion of women, No./total No. of inhabitants	INSEE	2019
Proportion of population aged <5 y, No./total No. of inhabitants	INSEE	2019
Proportion of population aged >65 y, No./total No. of inhabitants	INSEE	2019
Deprivation index^b^		
Median household income, disposable income per consumption unit multiplied by No. of people in the household/No. of people in the household	Observatory of territories	2017
Manual labor workers, No./total labor force (aged >15 y)	Observatory of territories	2017
High school graduates, No./No. of people not at school aged >15 y	INSEE	2017
Unemployment rate, No. unemployed/total labor force (aged 15-64 y)	INSEE	2017
**Living conditions**		
Overcrowded households, %^c^	Observatory of territories	2017
Mean No. of household members	Observatory of territories	2017
Travel by public transportation from residence to work, %	Observatory of territories	2017
Density of day care centers, No./total area	CAF	2018
Dogs per person, No./total No. of inhabitants aged >19 y	I-CAD	2020
**Agriculture and environment**		
Surface area of agricultural land/total area	Corine Land Cover	2018
Surface area of water/total area	Corine Land Cover	2018
Cattle density, No. of cattle/total area	Agreste: Ministerial Statistical Service for Agriculture	2015-2019
Pig density, No. of pig farms/total area	Agreste: Ministerial Statistical Service for Agriculture	2010
Poultry density, No. of poultry/total area	Agreste: Ministerial Statistical Service for Agriculture	2010
Sheep density, No. of sheep farms/total area	Agreste: Ministerial Statistical Service for Agriculture	2010

Abbreviations: CAF, family allowance funds; DDD, daily defined dose; DREES, Directorate of Research, Studies, Evaluations and Statistics; FINESS, National Database of Health Care Facilities; I-CAD, identification of domestic animals; INSEE, French National Institute for Statistics and Economic Research; SAE, Annual Statistics on Healthcare Facilities; 2GC, second-generation cephalosporins; 3GC, third-generation cephalosporins; 4GC, fourth-generation cephalosporins.

^a^ Surface area and total area were measured in square kilometers.

^b^ Indicates first main component of the principal component analysis of the 4 variables.

^c^ Defined as at least 1 room lacking compared with standard definition as follows: 1 living room; 1 room for each person in a family; 1 room for nonsingle nonfamily persons or 1 room for single persons 19 years or older; and 1 room for 2 children if they are of the same sex or are younger than 7 years and otherwise, 1 room per child.

Data regarding living conditions included the mean number of household members, overcrowding of principal residences, the proportion of individuals using public transportation between residence and work,^22^ the number of places in collective child care per square kilometer,^23^ and the rate of dog ownership in the population older than 19 years .^24^ The agrienvironmental data included the density in heads of cattle per square kilometer from 2015 to 2019; the density of poultry, pigs, and sheep in 2010^25^; and the surface area of water and agricultural land in square kilometers.^26^

### Statistical Analysis

Data were analyzed from January 1 to December 31, 2021. The variables are reported as either mean (SD) or median (IQR) based on the distribution of the respective explanatory variable for each department. The deprivation index was established using a previously described method.^17^ The index aims to provide a geographic indicator for the general population of social disadvantage specifically adapted to health studies on the French population, combining material and social disadvantages (eg, educational level, household income). A generalized linear model on count data following a Poisson distribution was used to analyze the association between the numbers of ESBL-producing *E coli* isolated from urine samples and the explanatory variables. The validity conditions of the Poisson regression model (independence, distribution, proportion of zero, and dispersion of responses) were checked. Data regarding the numbers of ESBL-producing *E coli* infections were independent, nonnormally distributed, and without inflation to zero. The overdispersion of the data for all variables required the use of a quasi-Poisson distribution. The variability of the number of strains tested in each department was considered by integrating an offset parameter in the generalized linear model with the number of *E coli* strains tested for their resistance to third-generation cephalosporins as reference. To fit the model, the effects of explanatory variables with *P* < .20 in the bivariate analysis were evaluated by a correlation matrix to avoid potential collinearity issues (eFigure 2 in Supplement 1). When the correlation between 2 variables was greater than 0.85, a choice was made between the 2 variables to be included in the multivariable model. A stepwise backward selection method was used to build the model using the Akaike information criterion for quasi-Poisson models. Multicollinearity of the final model was checked by calculating the variance inflation factor and by looking at the residuals graphically. Significance was set at 5%. Statistical analyses were performed with R, version 4.0.5 (Comprehensive R Archive Network).

## Results

Fifty-nine of 96 French metropolitan administrative departments were included in the analysis. The 37 remaining departments were excluded owing to the absence of participating clinical laboratories (n = 34) or because they did not reach the minimum number of samples (n = 3). Among the urine samples of individuals living in the 59 French departments included in the analysis in 2019, 444 281 *E coli* isolates were identified (range, 336-29 065 isolates per department) (Figure, A). The median age of individuals was 63 (IQR, 42-76) years; 48.5% were men and 51.5% were women. A total of 13 352 ESBL-producing *E coli* strains was identified (range, 6-958 per department) (Figure, B), for an overall 3.0% rate of ESBL-producing *E coli* (range, 1.4%-8.8% per department) (Figure, C) (see also eTable 1 in Supplement 1).

**Figure.**
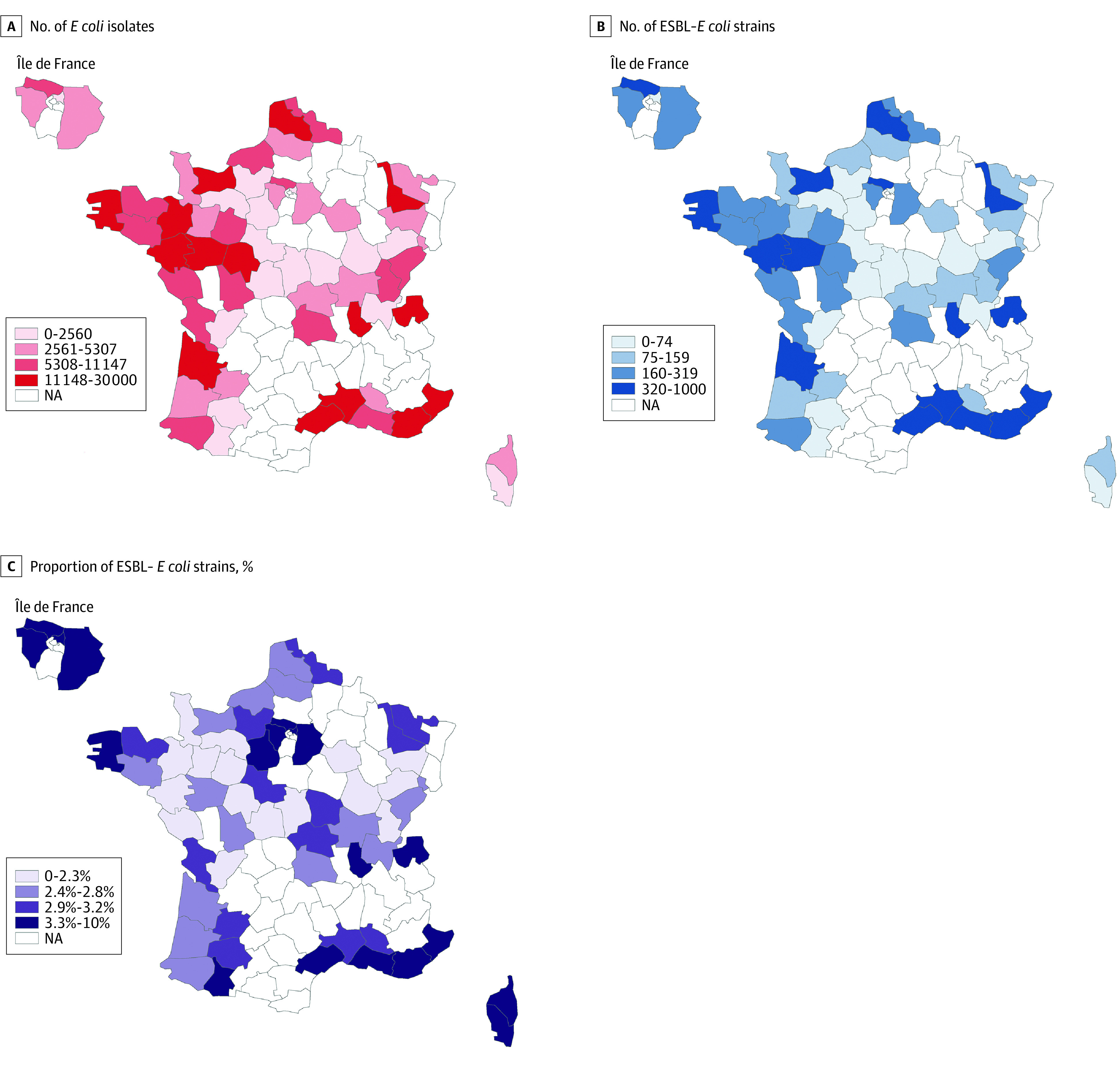
Extended-Spectrum β-Lactamase (ESBL)–Producing *Escherichia coli* Isolates From Urine Samples of Individuals With Community-Acquired Urinary Tract Infections Data are from January 1 to December 31, 2019, by French departments. NA indicates not available.

The ecological characteristics of administrative departments are displayed in Table 2. In bivariate analysis, the number of community-acquired ESBL-producing *E coli* UTIs was positively associated with the total consumption of antibiotics (β_1_ coefficient, 0.0002; *P* < .001), but also the consumption of β-lactams (β_1_ coefficient, 0.0003; *P* = .001), associations of penicillins (β_1_ coefficient, 0.001; *P* = .001), macrolides (β_1_ coefficient, 0.001; *P* < .001), fluoroquinolones (β_1_ coefficient, 0.002; *P* < .001), tetracyclines (β_1_ coefficient, 0.001; *P* = .03), and sulfonamides and trimetroprim (β_1_ coefficient, 0.004; *P* = .04) (eTable 2 in Supplement 1). The same association was found with the densities of hospital beds (β_1_ coefficient, 0.042; *P* = .001), nursing homes (β_1_ coefficient, 0.002; *P* = .004), and antibiotic prescribers (β_1_ coefficient, 0.053; *P* < .001). Regarding sociodemographic characteristics, the proportion of women (β_1_ coefficient, 0.266; *P* = .004) and proportion of the population younger than 5 years (β_1_ coefficient, 0.171; *P* = .02) were positively associated with the number of ESBL-producing *E coli* infections. Among living conditions, a positive association was found with the proportion of overcrowded households (β_1_ coefficient, 0.084; *P* < .001), individuals traveling by public transportation (β_1_ coefficient, 0.021; *P* < .001), the density of day care centers for children (β_1_ coefficient, 0.023; *P* = .003), and the proportion of dogs per persons older than 19 years (β_1_ coefficient, −0.027; *P* = .003). The percentage of agricultural land (β_1_ coefficient, −0.008; *P* < .001) and cattle density (β_1_ coefficient, −0.006; *P* < .001) were negatively associated, whereas the proportion of water surface area (β_1_ coefficient, 0.082; *P* = .004) was positively associated with the number of ESBL-producing *E coli* isolates.

**Table 2.** Characteristics of the French Administrative Departments Analyzed

Characteristic	Values^a^
**Health care–related **	
Antibiotic consumption, DDDs/1000 inhabitants/y	
All	8373 (811)
β-Lactams	4616 (421)
Broad-spectrum penicillin	2900 (287)
Penicillin associations	1595 (222)
Cephalosporins	640 (136)
2GC	140 (43)
3GC and/or 4GC	495 (103)
Macrolides	1077 (162)
Fluoroquinolones	504 (84)
Sulfonamides and trimethoprim	144 (26)
Tetracyclines	982 (178)
Associations and others	399 (63)
Density of antibiotic prescribers, No./100 000 inhabitants	188.3 (50.6)
Density of hospital beds, median (IQR), No. of beds per square kilometer	0.6 (0.4 to 1.0)
Density of nursing homes, median (IQR), No. of homes per square kilometer	1.3 (0.8 to 1.9)
**Sociodemographic **	
Population density, median (IQR), No. of inhabitants per square kilometer	99.1 (59.1 to 6966.2)
Proportion of women, %	51.5 (0.4)
Proportion of population aged <5 y, %	5.2 (0.8)
Proportion of population aged >65 y, %	22.2 (3.8)
Deprivation index, median (IQR), %	0.1 (−0.4 to 0.4)
Household income, median per consumption unit	20 756 (1377)
Manual labor workers, %	13.4 (2.1)
High school graduates, %	17.2 (1.3)
Unemployment rate, %	13.3 (2.3)
**Living conditions**	
Overcrowded households, median (IQR), %	2.0 (1.5 to 2.9)
No. of household members	2.2 (0.1)
Travel by public transportation, median (IQR), %	4.9 (2.8 to 9.4)
Density of day care centers, median (IQR), No. per square kilometer	0.4 (0.2 to 1.1)
Dogs per person aged >19 y, %	21.6 (6.1)
**Agriculture and environment **	
Agricultural land, median (IQR), %	63.6 (46.2 to 79.1)
Surface water, median (IQR), %	0.7 (0.3 to 1.0)
Cattle density, median (IQR), No. per square kilometer	30.9 (8.4 to 56.1)
Pig density, median (IQR), No. per square kilometer	7.9 (2.9 to 19.9)
Poultry density, median (IQR), No. per square kilometer	94.4 (21.9 to 292.4)
Sheep density, median (IQR), No. per square kilometer	5.2 (3.2 to 9.0)

Abbreviations: DDD, daily defined dose; 2GC, second-generation cephalosporins; 3GC, third-generation cephalosporins; 4GC, fourth-generation cephalosporins.

^a^ Unless otherwise indicated, data are expressed as mean (SD).

Seven covariates were associated with the number of community-acquired ESBL-producing *E coli* UTIs in the multivariate analysis (Table 3). None of these variables had a variance inflation factor of greater than 5, validating the absence of multicollinearity. Of these, the consumption of fluoroquinolones (adjusted β coefficient, 0.002 [95% CI, 0.001-0.002]; *P* < .001) and tetracyclines (adjusted β coefficient, 0.0002 [0.00004 to 0.00039]; *P* = .02), the percentage of people younger than 5 years (adjusted β coefficient, 0.112 [95% CI, 0.040-0.185]; *P* = .004), overcrowded households (adjusted β coefficient, 0.049 [95% CI, 0.034 to 0.062]; *P* < .001), and poultry density (adjusted β coefficient, 0.0001 [95% CI, 0.0001-0.0002]; *P* < .001) were positively associated with the number of community-acquired ESBL-producing *E coli* UTIs. The deprivation index (adjusted β coefficient, −0.115 [95% CI, −0.165 to −0.064]; *P* < .001) and the proportion of water surface area (adjusted β coefficient, −0.052 [−0.081 to −0.024]; *P* = .001) were negatively associated with the number of community-acquired ESBL-producing *E coli* UTIs. Interpretation of the multivariate model using the parameters from a single department is shown in Table 4.

**Table 3.** Multivariate Regression Analysis of French Administrative Department Characteristics Associated With Community-Acquired Extended-Spectrum β-Lactamase–Producing *Escherichia coli* Urinary Tract Infections

Characteristic	Adjusted β_1_ (95% CI)	*P* value
Health care–related		
Fluoroquinolones consumption	0.002 (0.001 to 0.002)	<.001
Tetracycline consumption	0.0002 (0.00004 to 0.00039)	.02
Sociodemographic		
Proportion of people aged <5 y	0.112 (0.040 to 0.185)	.004
Deprivation index	−0.115 (−0.165 to −0.064)	<.001
Living conditions		
Overcrowded households	0.049 (0.034 to 0.062)	<.001
Agriculture and environment		
Water surface area	−0.052 (−0.081 to −0.024)	.001
Poultry density	0.0001 (0.0001 to 0.0002)	<.001

**Table 4.** Interpretation of the Multivariate Model Using the Parameter of a Single Department^a^

Scenario	Regression coefficient, β_1_	Extra cases of community-acquired ESBL-producing *Escherichia coli* UTIs	
		Multiplicative factor, % increase	Crude No. of additional ESBL-producing *E coli* UTIs
Increased quinolone consumption of 100 DDDs/1000 inhabitants/y	0.002	0.02^b^	0.09
Increased tetracycline consumption of 100 DDDs/1000 inhabitants/y	0.0002	0.002^c^	0.009
Increase of 1% in population aged <5 y	0.112	11.85^d^	53.1
Increase of 1% in overcrowded households	0.049	5.0^e^	22.4
Additional poultry flock ≥10 000 heads	0.0001	0.014^f^	0.06
Increase of 1% in water surface area	−0.052	−5.07^g^	−22.7
Increase of 0.5 in deprivation index	−0.115	−5.59^h^	−25.03

Abbreviations: DDD, daily defined dose; ESBL, extended-spectrum β-lactamase; UTI, urinary tract infection.

^a^ The Loire-Atlantique department is considered a reference with a surface area of 6.874.4 km^2^, a total population of 1 427 913 inhabitants (5.8% of whom are younger than 5 years), an annual number of *E coli* UTIs of 22 610, and a rate of ESBL-producing *E coli* UTIs of 2.2%. The number of cases of ESBL-producing *E coli* UTI was calculated as 22 610 × 0.0216 = 448 cases per year. The expected number of additional cases of ESBL-produced *E coli* UTI is presented for different scenarios.

^b^ Calculated as e^0.002 × (100/1000)^ – 1 = 0.0002.

^c^ Calculated as e^0.002 × (100/1000)^ – 1 = 0.00002.

^d^ Calculated as e^0,112^ − 1 = 0.1185.

^e^ Calculated as e^0,049^ − 1 = 0.05.

^f^ Calculated as e^0.0001 × (10 000/6874.4)^ – 1 = 0.00014.

^g^ Calculated as e^−0,052^ − 1 = −0.0507.

^h^ Calculated as e^−0,115 × 0,5^ − 1 = −0.0559.

## Discussion

In this study, we used population-level human, agricultural, and environmental characteristics to assess the association between ecological factors and the number of community-acquired ESBL-producing *E coli* UTIs throughout administrative departments of metropolitan France in 2019. Community-acquired ESBL-producing *E coli* UTIs were associated with the local percentage of children younger than 5 years, overcrowded households, human consumption of fluoroquinolones and tetracyclines, and poultry density.

Previous studies^4^ have described antibiotic therapies, mainly β-lactams and fluoroquinolones, as an important risk factor for ESBL-producing *E coli* colonization and infections. The positive association of community-acquired ESBL-producing *E coli* UTIs with fluoroquinolones confirms the importance of efforts to reduce their consumption. Despite the known broad spectrum of this antibiotic class, the association of tetracycline consumption with the spatial distribution of community-acquired ESBL-producing *E coli* UTIs was not expected. Cephalosporins and tetracycline were previously ranked as the highest monotherapies in promoting ESBL colonization during hospitalization.^27^ The tetracyclines may have the same ability in selecting ESBL colonization in the community setting with an increased risk of subsequent infections with these resistant strains. The high proportion of tetracycline use among the total consumption of antibiotics in primary care in France (15.6% and third position of the most consumed antibiotic classes in 2020) calls for awareness regarding the stewardship of this antibiotic class.^28^

The mechanisms of human-to-human transmission of ESBL-producing *E coli* in the community are not well understood. The positive associations between community-acquired ESBL-producing *E coli* UTI and the population of individuals younger than 5 years or overcrowded households highlight potential areas of interest for the management of the antimicrobial resistance in the community. Approximately two-thirds of carriage of community-acquired ESBL-producing *E coli* has been attributed to human-to-human transmission, with the nonhuman sources (food, animal, and environmental) accounting for the other third.^29^ In a study performed in London,^7^ the community-level overcrowding rate was associated with ESBL rectal carriage at hospital admission. The household may play an important role in the spread of ESBL-producing *E coli* owing to the proximity of contacts, the sharing of similar exposures, and multiple opportunities for cross-transmission. Members of households with preschool-aged children have been previously found to be particularly exposed to an increased risk of intestinal carriage of ESBL and AmpC β-lactamase.^30^ The high exposure of children younger than 5 years to antibiotics^31^ combined with their attendance in day care centers may contribute to the spread of ESBL-producing *E coli*, increasing the risk of infection at the population level. In our study, preschool day care centers were positively associated with the number of ESBL-producing *E coli* isolates in univariate analysis but not after adjustment of the model. A cohort study assessing the evolution of and factors associated with the ESBL carriage among households with preschool-aged children may improve the understanding of the global ESBL epidemiology.

We found an association between the density of poultry and the occurrence of ESBL-producing *E coli* in urine samples. The presence of ESBL-producing bacteria in retail chicken meat has been documented repeatedly.^32^ Similar ESBL-producing *E coli* found from chicken meat and from humans makes chicken meat a plausible source of ESBL-producing *E coli* in humans. However, the mechanism of poultry-to-human transmission remains controversial, and the epidemiological association is still poorly understood.^33^ In France, poultry are mostly raised in indoor systems and largely exposed to antibiotics. In 2019, the animal-level exposure to antimicrobials in poultry was 0.396 (ie, 39.6% of the total mass of poultry was treated with antibiotics).^34^ Despite a dramatic decrease of antimicrobial use in farm-raised animals,^34^ our results suggest the need to maintain the efforts with a particular attention to poultry and the production methods. Initially positively associated with community-acquired ESBL-producing *E coli* UTIs in bivariate analysis, water surface area was found to be negatively associated in the final model. The percentage of water surface area was used to assess the previously described association of community-acquired ESBL-producing *E coli* UTIs with recreational freshwater swimming.^35^ The shift observed from bivariate to multivariable models might be owing to the adjustment on sociodemographic characteristics and living conditions. In departments with overcrowded households, a high proportion of people younger than 5 years, and a low deprivation index, mostly corresponding to highly urbanized areas, we assume that people are less likely to swim in surface water. Moreover, this variable is probably more reflective of the density of population in French departments than the risk linked to wastewater, potentially explaining the negative association found in the present study.

The literature is discordant regarding the association between deprivation and the risk of infections by antimicrobial-resistant bacteria in the community. As in previously published studies,^2,36^ the poverty identified by the deprivation index was negatively associated with the presence of ESBL-producing *E coli*. Other studies^5^ assumed a role of socioeconomic factors such as a low level of community adult education in estimating ESBL colonization. A recent ecological analysis in Chicago^3^ found an association between resistant *Enterobacterales* infections and the percentage of uninsured residents but not the percentage of households beneath the poverty line. Such a discrepancy may reflect the multidimensional aspect of poverty and the difficulty of estimating this factor using population-level variables.

### Limitations

Our findings are limited by the study design. Because we used an ecological approach, the results should be interpreted with caution, especially patient-related factors (age, sex, antibiotic consumption, and deprivation index). We used data either during the same period or averaged during previous years (eg, antibiotic consumption from 2015 to 2019) to consider the latency period that may exist between exposure and the occurrence of resistance. Urine samples were the only source of infection included in this investigation. In 2019, 98.8% of the 565 483 sample with positive findings for *Enterobacterales* collected through the PRIMO surveillance system were urine samples.^37^ Administrative departments were used as boundaries of the geographic areas. Finally, microbiological data were not available for 37 of the 96 administrative departments of metropolitan France. Clinical laboratories participate in the PRIMO surveillance system on a voluntary basis, with a growing participation from year to year. Although the network in 2019 did not comprehensively cover the French metropolitan territory, the choice was made to use only data for the full year 2019 to include the largest sample of departments in the study. We acknowledge that this fairly small number of administrative departments may have influenced the model outputs. Nevertheless, our findings corroborate previous investigations^4^ that have identified important department-level variations in community-acquired ESBL-producing *E coli* UTI risk in association with demographic, living conditions, health care, agricultural, and environmental factors.

## Conclusions

The findings of this cross-sectional study suggest that multiple factors are associated with the occurrence of community-acquired ESBL-*E coli* UTI and confirm the complicated epidemiology of ESBL-producing *Enterobacteriaceae*. These findings also suggest associations among human health, animal health, and environmental factors and the importance of combined cross-sectoral strategies for surveillance and prevention of ESBL-producing *E coli* infections that follow the One Health approach. Additional research is needed to explore the determinants of the transmission of ESBL among household members.

## Notes

Supplement 1. **eFigure 1.** Spatial Distribution of the 1013 Clinical Laboratories That Participated in the PRIMO Surveillance System in 2019 **eFigure 2.** Results of the Correlation Matrix Performed to Explore the Multicollinearity of Variables **eTable 1.** Number and Percentages of *Escherichia coli* and Extended-Spectrum β-Lactamase–Producing *E coli* Strains Included in the Study According to French Departments in 2019 **eTable 2.** French Department Characteristics Significantly Associated With Presence of Extended-Spectrum β-Lactamase–Producing *Escherichia coli* Community-Acquired Urinary Tract Infections, Results of Bivariate Analysis

Supplement 2.
Nonauthor Collaborators
