# Supplementary material for: Assessment of Factors Associated With Community-Acquired Extended-Spectrum β-Lactamase–Producing Escherichia coli Urinary Tract Infections in France
Source: JAMA Netw Open. 2022 Sep 21;5(9):e2232679. doi: 10.1001/jamanetworkopen.2022.32679 (PMC9494187; doi:10.1001/jamanetworkopen.2022.32679)
Supplement: Supplement 1. — eFigure 1. Spatial Distribution of the 1013 Clinical Laboratories That Participated in the PRIMO Surveillance System in 2019 eFigure 2. Results of the Correlation Matrix Performed to Explore the Multicollinearity of Variables eTable 1. Number and Percentages of Escherichia coli and Extended-Spectrum β-Lactamase–Producing E coli Strains Included in the Study According to French Departments in 2019 eTable 2. French Department Characteristics Significantly Associated With Presence of Extended-Spectrum β-Lactamase–Producing Escherichia coli Community-Acquired Urinary Tract Infections, Results of Bivariate Analysis [file jamanetwopen-e2232679-s001.pdf]

Supplementary Online Content

Paumier A, Asquier-Khati A, Thibaut S, et al; French Clinical Laboratories Nationwide Network. Assessment of factors associated with community-acquired extended-spectrum β-lactamase–producing *Escherichia coli* urinary tract infections in France. *JAMA Netw Open*. 2022;5(9):e2232679. doi:10.1001/jamanetworkopen.2022.32679

eFigure 1. Spatial Distribution of the 1013 Clinical Laboratories That Participated in the PRIMO Surveillance System in 2019

eFigure 2. Results of the Correlation Matrix Performed to Explore the Multicollinearity of Variables

eTable 1. Number and Percentages of *Escherichia coli* and Extended-Spectrum β-Lactamase–Producing *E coli* Strains Included in the Study According to French Departments in 2019

eTable 2. French Department Characteristics Significantly Associated With Presence of Extended-Spectrum β-Lactamase–Producing *Escherichia coli* Community-Acquired Urinary Tract Infections, Results of Bivariate Analysis

This supplementary material has been provided by the authors to give readers additional information about their work.

© 2022 Paumier A et al. *JAMA Network Open*.

eFigure 1. Spatial Distribution of the 1013 Clinical Laboratories That Participated in the PRIMO Surveillance System in 2019

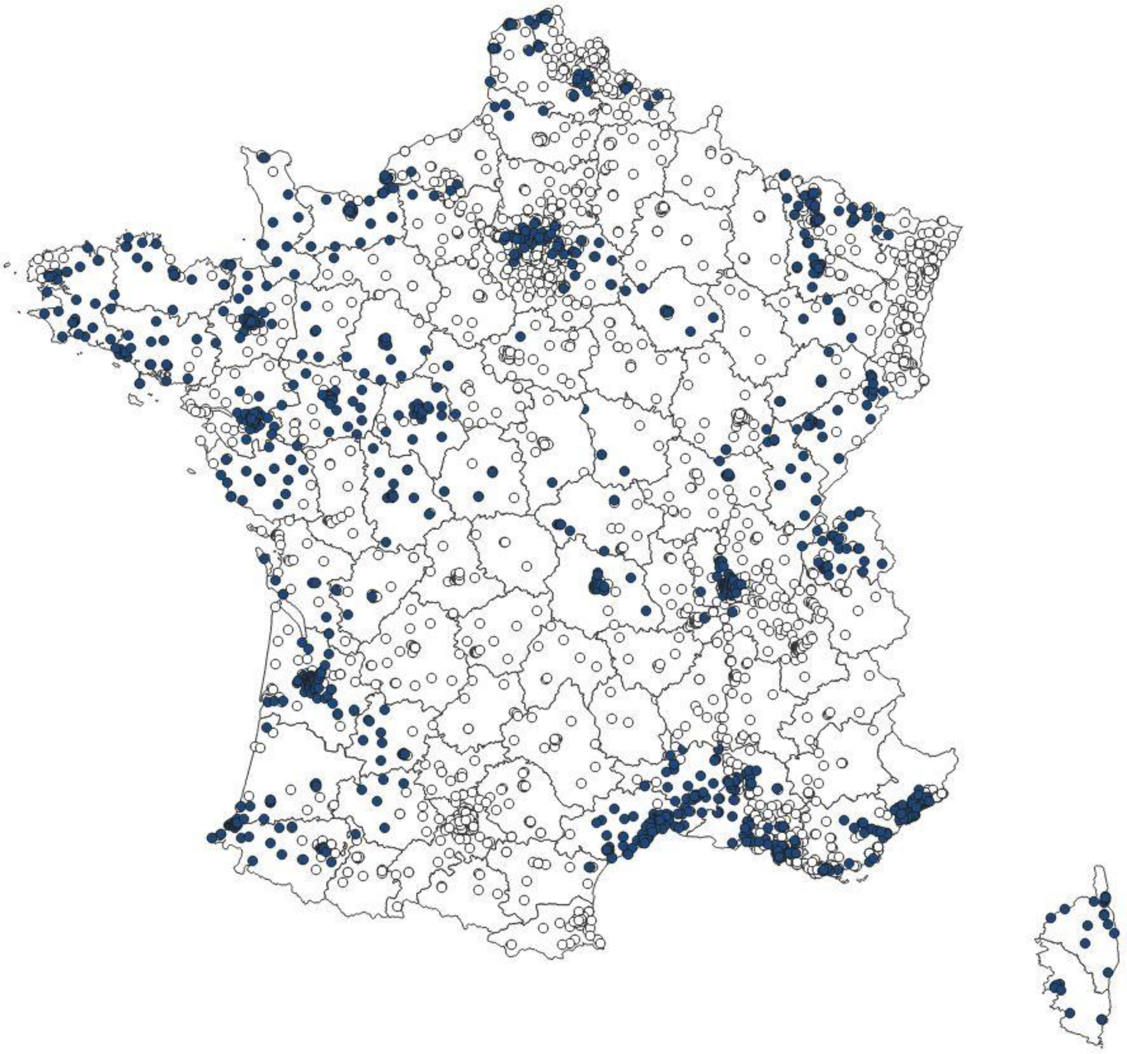

Blue dotes: Participating clinical laboratories; Blank dotes: Clinical laboratories in activity in 2019 but not participating to the PRIMO surveillance system.

© 2022 Paumier A et al. *JAMA Network Open*.

eFigure 2. Results of the Correlation Matrix Performed to Explore the Multicollinearity of Variables

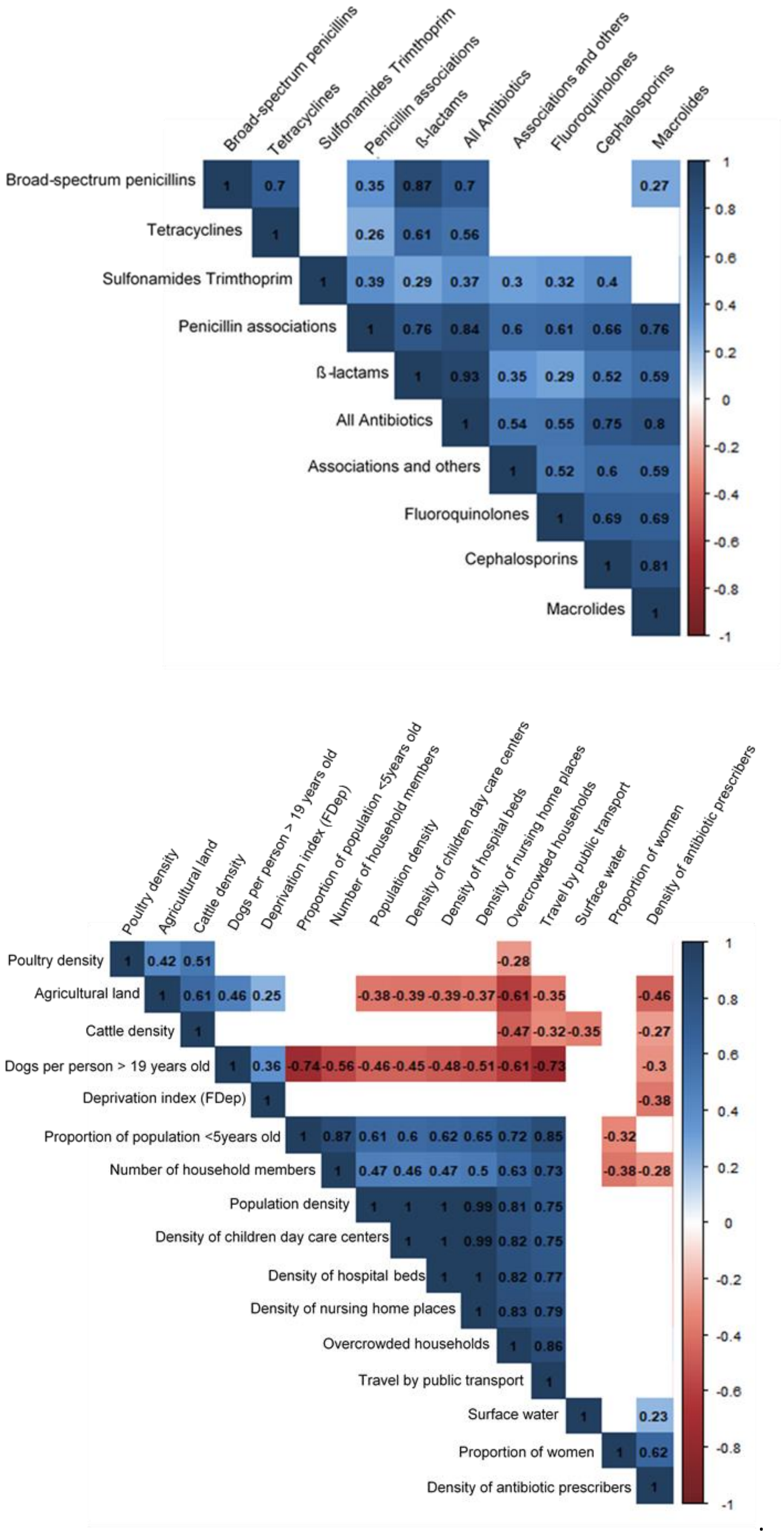

© 2022 Paumier A et al. *JAMA Network Open*.

eTable 1. Number and Percentages of *Escherichia coli* and Extended-Spectrum β-Lactamase–Producing *E coli* Strains Included in the Study According to French Departments in 2019

French departments	Number of ESBL-*E.coli*	Number of *E.coli*	Proportion of ESBL-*E.coli* (%)
Ain	32	1,217	2.63
Allier	105	3,386	3.10
Alpes-Maritimes	958	20,919	4.58
Aube	85	4,021	2.11
Bouches-du-Rhône	638	7,545	8.46
Calvados	458	18,806	2.44
Charente	22	999	2.20
Charente-Maritime	257	8,674	2.96
Cher	6	412	1.46
Corse-du-Sud	37	951	3.89
Côte-d'Or	17	1,196	1.42
Côtes-d'Armor	188	6,404	2.94
Doubs	296	10,961	2.70
Eure	48	1,614	2.97
Eure-et-Loir	23	632	3.64
Finistère	479	14,526	3.30
Gard	380	13,191	2.88
Gers	29	913	3.18
Gironde	540	20,801	2.60
Haute-Corse	128	3,404	3.76
Haute-Saône	43	2,487	1.73
Haute-Savoie	387	11,933	3.24
Hautes-Pyrénées	14	336	4.17
Hérault	938	29,064	3.23
Ille-et-Vilaine	303	13,338	2.27
Indre	12	701	1.71
Indre-et-Loire	286	13,164	2.17
Jura	158	7,082	2.23
Landes	82	3,177	2.58
Loir-et-Cher	17	552	3.08
Loire-Atlantique	490	22,610	2.17
Lot-et-Garonne	80	2,634	3.04
Maine-et-Loire	350	15,176	2.31
Manche	117	5,272	2.22
Mayenne	84	3,930	2.14
Meurthe-et-Moselle	720	22,747	3.17
Morbihan	168	7,282	2.31
Moselle	159	5,280	3.01
Nièvre	71	2,379	2.98
Nord	256	8,883	2.88
Orne	12	738	1.63
Pas-de-Calais	335	12,126	2.76
Puy-de-Dôme	272	9,844	2.76
Pyrénées-Atlantiques	174	7,404	2.35
Rhône	396	11,333	3.49
Saône-et-Loire	119	4,723	2.52
Sarthe	211	9,712	2.17
Seine-et-Marne	220	5,307	4.15
Seine-Maritime	149	5,323	2.80
Seine-Saint-Denis	58	893	6.49
Somme	85	3,639	2.34
Territoire de Belfort	78	2,858	2.73

© 2022 Paumier A et al. *JAMA Network Open*.

Val-d'Oise	379	6,797	5.58
Var	528	12,078	4.37
Vaucluse	156	5,018	3.11
Vendée	190	8,381	2.27
Vienne	214	9,114	2.35
Vosges	119	5,246	2.27
Yvelines	196	5,148	3.81

© 2022 Paumier A et al. *JAMA Network Open*.

eTable 2. French Department Characteristics Significantly Associated With Presence of Extended-Spectrum β-Lactamase–Producing *Escherichia coli* Community-Acquired Urinary Tract Infections, Results of Bivariate Analysis

Characteristics	Coefficient β_1	*p*-value
**Healthcare-related characteristics**		
All Antibiotics	0.0002	0.001
ß-lactams	0.0003	0.004
Broad-spectrum penicillins	0.0003	0.15
Penicillin associations	0.001	0.001
Cephalosporins	0.0005	0.16
2GC consumption	0.002	0.20
3GC/4GC consumption	0.0005	0.26
Macrolides consumption	0.001	<0.001
Fluoroquinolones	0.002	<0.001
Sulfonamides Trimethoprim	0.004	0.04
Tetracyclines	0.001	0.03
Associations and others	0.001	0.11
Density of antibiotic prescribers	0.053	<0.001
Density of hospital beds	0.042	0.001
Density of nursing home places	0.002	0.004
**Socio-demographic characteristics**		
Population density	0.0002	0.005
Proportion of women	0.266	0.004
Proportion of population <5years old	0.171	0.02
Proportion of population >65 years old	-0.009	0.551
Deprivation index (FDep)	-0.111	0.18
**Living conditions**		
Overcrowded households	0.084	<0.001
Number of household members	0.776	0.07
Travel by public transport	0.021	<0.001
Density of children day care centers	0.023	0.003
Dogs per person > 19 years old	-0.027	0.003
**Agriculture and Environment characteristics**		
Agricultural land	-0.008	<0.001
Surface water	0.082	0.004
Cattle density	-0.006	<0.001
Pig density	-0.0004	0.50
Poultry density	-0.0001	0.1
Sheep density	0.004	0.31

Abbreviations: 2GC, second generation cephalosporins; 3GC, third generation cephalosporins; 4GC, fourth generation cephalosporins; km^2 , square kilometer.

© 2022 Paumier A et al. *JAMA Network Open*.
