# Supplementary material for: Assessment of Factors Associated With Community-Acquired Extended-Spectrum β-Lactamase–Producing Escherichia coli Urinary Tract Infections in France
Source: JAMA Netw Open. 2022 Sep 21;5(9):e2232679. doi: 10.1001/jamanetworkopen.2022.32679 (PMC9494187; doi:10.1001/jamanetworkopen.2022.32679)
Supplement: Supplement 2. — Nonauthor Collaborators [file jamanetwopen-e2232679-s002.pdf]

Supplemental Online Content: Nonauthor Collaborators
*First name, last name, and suffix (if applicable) are required and will appear in PubMed.

*Group Name(s): French Clinical Laboratories Nationwide Network							
***First Name and Middle Initial(s)**	***Last Name**	***Suffix (eg, Jr, III)**	Academic Degrees	Institution	Location (city, state/province, country)	Role or Contribution, eg, chair, principal investigator	Group (if more than 1 Group listed in the byline) and/or Subgroup (eg, Steering Committee)
Sophie	Poyet		MD	DYOMEDEA	Lyon , France	data collection	
Guillaume	Deléglise		PharmD	GENBIO	Clermont Ferrand, France	data collection	
Lydie	Libier		PharmD	AX BIO OCEAN	Bayonne, France	data collection	
Simon	Benzimra		PharmD	BIOLAB33	Le Haillan, France	data collection	
Denis	Laforest		MD	BIOCENTRE	Coutances, France	data collection	
Laurent	Clotteau		PharmD	BIOLOR	Lorient, France	data collection	
Karine	Michez		MD	OCEALAB	Vannes, France	data collection	
Marie-Jeanne	Blanc-Galiby		PharmD	BLANC-GALIBY	Hennebont, France	data collection	
Franck	Kerdavid		PharmD	ALLIANCE ANABIO	Melesse, France	data collection	
Brigitte	Coude Du Foresto		MD	BIOLIANCE	Nantes, France	data collection	
François	Maillet		MD	BIOLOIRE	Nantes, France	data collection	
Hubert	Banctel		PharmD	SBL BIO	Saint Brieuc, France	data collection	
Jean	Lacroze		PharmD	BIOARVOR	Lannion , France	data collection	
Marie-pierre	Thibault,		PharmD	BIOPOLE	Loudéac , France	data collection	
Eric	Grandsire		PharmD	DYNALAB	Romilly sur Seine, France	data collection	
Pierre-Yves	Léonard		MD	LABORIZON MAINE ANJOU	Le Mans, France	data collection	
Jean-Francois	Culard		MD	ANALYSIS 88	Epinal , France	data collection	
Anne	Holstein		PharmD	ABO +	Tours , France	data collection	
Beatrice	Dubet		PharmD	LBM DUBET	Neuville aux bois, France	data collection	
Sebastien	Fougnot		MD	ATOUTBIO	Nancy , France	data collection	
Bruno	Guesnon		PharmD	BIORANCE	Saint Malo , France	data collection	
Anne-Sophie	Reinhard		PharmD	BIOCELIANDE	Montauban de Bretagne, France	data collection	
Jean Philippe	Rault		PharmD	ESPACEBIO	Metz , France	data collection	
Brieuc	Gestin		PharmD	LABAZUR	Chateaulin , France	data collection	

Supplemental Online Content: Nonauthor Collaborators
*First name, last name, and suffix (if applicable) are required and will appear in PubMed.

*First Name and Middle Initial(s)	*Last Name	*Suffix (eg, Jr, III)	Academic Degrees	Institution	Location (city, state/province, country)	Role or Contribution, eg, chair, principal investigator	Group (if more than 1 Group listed in the byline) and/or Subgroup (eg, Steering Committee)
Jerome	Fleurance		MD	ISOSEL	Ancenis , France	data collection	
Alisson	Vrain		PharmD	ANDEBIO	Angers , France	data collection	
Philippe	Andorin		PharmD	BIOLARIS	Laval, France	data collection	
Géoffroy	De Gastines		MD	BIORYLIS	La Roche sur Yon , France	data collection	
Christine	Fantinato		PharmD	SEVRE BIOLOGIE	Les Herbiers, France	data collection	
Vidal	Plong		PharmD	ACTIV’BIOLAB	Challans, France	data collection	
Nicolas	Le Moing		PharmD	RESEAUBIO	La Chapelle sur Erdre, France	data collection	
Emilie	Pradier		PharmD	CARMES	Caen, France	data collection	
Regis	Gouarin		PharmD	BIONACRE	Caen, France	data collection	
Didier	Grisard		PharmD	LBM FLERS & CONDE	Flers , France	data collection	
Stéphanie	Arsene		PharmD	CERBALLIANCE NORMANDIE	Lisieux, France	data collection	
Elodie	Jobert		PharmD	MIRIALIS	Annecy , France	data collection	
Charles	Veron		MD	LBM CCF	Ajaccio , France	data collection	
Gérard	Payro		PharmD	CERBALLIANCE-CHARENTE	Saintes, France	data collection	
Annie	Allery		PharmD	BIO86	Poitiers , France	data collection	
Helene	Valade		PharmD	BIOFFICE	Bordeaux, France	data collection	
Delphine	Boraud		MD	EXALAB Groupe LABEXA	Le Haillan , France	data collection	
Remi	Gebeile		PharmD	DYNABIO	Lyon , France	data collection	
Elisabeth	Parisi		PharmD	VIALLE	Bastia, France	data collection	
Francoise	Alluin		PharmD	2A2B	Porto Vecchio , France	data collection	
Caroline	Coulon		PharmD	BIOAXIOME	Avignon , France	data collection	
Guillaume	Teissier		MD	LABOSUD	Montpelier , France	data collection	
Arnaud	François		PharmD	BIOESTEREL	Mandelieu-la-Napoule, France	data collection	
Gilles	Defrance		PharmD	BIOFUTUR	L’isle Adam, France	data collection	
Gisele	Gay		PharmD	LABOSUD PROVENCE	Marseille , France	data collection	
Olivier	Duquesnoy		PharmD	BIOPATH	Dunkerque, France	data collection	
Fabienne	Artur		PharmD	BIOCEANE	Le Havre, France	data collection	
Sylvain	Millet		PharmD	MEDILYS	Dole, France	data collection	

Supplemental Online Content: Nonauthor Collaborators
*First name, last name, and suffix (if applicable) are required and will appear in PubMed.

***First Name and Middle Initial(s)**	***Last Name**	***Suffix (eg, Jr, III)**	Academic Degrees	Institution	Location (city, state/province, country)	Role or Contribution, eg, chair, principal investigator	Group (if more than 1 Group listed in the byline) and/or Subgroup (eg, Steering Committee)
Pierre	Marchenay		PharmD	LPA18	Vesoul , France	data collection	
Norbert	Desbiolles		MD	BIOGROUP-LCD	Montbéliard , France	data collection	
Marie Carole	Paolini		MD	BDCBM5	Besançon , France	data collection	
Eugénie	Mbenga		MD	BIOLAB	Beaune , France	data collection	
Arel	Desjardins		MD	EVORIAL	Nevers, France	data collection	
Christian	Ehret		PharmD	BIOLAB90	Belfort, France	data collection	
